# Prospective Investigation of Feline Leukemia Virus Infection in Stray Cats Subjected to a Trap–Neuter–Return Program in Switzerland

**DOI:** 10.3390/v16030394

**Published:** 2024-03-02

**Authors:** Marina L. Meli, Benita Pineroli, Esther Geisser, Regina Hofmann-Lehmann

**Affiliations:** 1 Clinical Laboratory, Department of Clinical Diagnostics and Services, and Center for Clinical Studies, Vetsuisse Faculty, University of Zurich, 8057 Zurich, Switzerland; mmeli@vetclinics.uzh.ch (M.L.M.);; 2 Network for Animal Protection (NetAP), 8133 Esslingen, Switzerland; info@netap.ch

**Keywords:** FeLV, retrovirus, prevalence, stray cats, free-roaming cats, RT-qPCR, virus shedding, virus reservoir, vaccination, veterinary sciences

## Abstract

Feline leukemia virus (FeLV) remains a serious concern in some countries despite advances in diagnostics and vaccines. FeLV-infected cats often have reduced lifespans due to FeLV-associated diseases. The infection is transmitted through social interactions. While Northern European countries have reported a decrease in FeLV among pet cats, Switzerland’s rates remain stagnant at 2.7% (2016/17: 95% CI 1.4–5.2%). Research on FeLV in Swiss stray cats has been lacking, even though these animals could serve as a virus reservoir. Sampling stray cats that do not receive regular veterinary care can be challenging. Collaboration with the Swiss Network for Animal Protection (NetAP) allowed for the prospective collection of saliva samples from 1711 stray cats during a trap–neuter–return program from 2019 to 2023. These samples were tested for FeLV RNA using RT-qPCR as a measure for antigenemia. Viral RNA was detected in 4.0% (95% CI 3.1–5.0%) of the samples, with 7.7% (95% CI 4.9–11.3%) in sick cats and 3.3% (95% CI 2.4–4.4%) in healthy ones. We identified three geographically independent hotspots with alarmingly high FeLV infection rates in stray cats (up to 70%). Overall, including the previous data of privately owned cats, FeLV-positive cats were scattered throughout Switzerland in 24/26 cantons. Our findings underscore welfare concerns for FeLV infections among stray cats lacking veterinary attention, highlighting the potential risk of infection to other free-roaming cats, including those privately owned. This emphasizes the critical significance of vaccinating all cats with outdoor access against FeLV and developing programs to protect cats from FeLV infections.

## 1. Introduction

Infection with feline leukemia virus (FeLV) can result in severe and often fatal illnesses in domestic cats. Progressive FeLV infections significantly diminish life expectancy and can lead to incurable conditions, including lymphoma, leukemia, non-regenerative and immune-mediated hemolytic anemia, and chronic or recurrent infections due to compromised immune function [1]. Our understanding of FeLV infections in client-owned cats in Switzerland has been well documented and shared with veterinarians [2,3,4]. For many years, FeLV prevalence decreased in Switzerland, like in many other countries, thanks to reliable tests, programs to segregate progressively infected cats, an understanding of FeLV pathogenesis, and the availability of effective vaccines [2,5,6]. However, since 2003, a stagnation in the prevalence of progressive infection has been recognized within the population of privately owned cats in Switzerland, constantly hovering around 2% [2,3].

When it comes to stray cats, we currently lack comprehensive data for Switzerland. Our knowledge is limited to isolated incidents and anecdotal reports suggesting the presence of FeLV issues in specific regions, without concrete data to support this. Obtaining information regarding the prevalence of FeLV in free-roaming cats in Switzerland is crucial, as this feline population could potentially serve as a challenging-to-manage reservoir for the virus. Therefore, data on FeLV infections in stray cats are vital for efforts aimed at reducing FeLV prevalence across Switzerland, following the successful strategies implemented in other European countries [3].

To assess the prevalence of FeLV, various methods and diagnostic techniques can be employed [4,7,8]. When it comes to identifying virus shedders, the epidemiological and clinical most important outcome of an FeLV infection, the simplest approach is to detect the virus p27 antigen in feline blood samples. This can be achieved through convenient point-of-care tests or sophisticated laboratory-based enzyme-linked immunosorbent assays. However, these methods require the collection of a blood sample, which may be invasive for the cat. As an alternative, a less intrusive approach involves the collection of saliva samples, in which virus shedders can be identified using highly sensitive reverse transcriptase polymerase chain reaction (RT-qPCR). Notably, the levels of viral RNA in saliva and FeLV p27 antigen in blood exhibit an almost perfect degree of agreement [9,10]. Furthermore, the collection of saliva samples offers the advantage of pooling and analyzing multiple samples without compromising the detection of even 1 in every 30 positive cases [10]. This novel methodology, involving the collection of saliva samples, pooling, and a subsequent RT-qPCR analysis, was successfully employed in the first pan-European FeLV study, encompassing 6005 cats across 32 countries [3].

The current study aimed to assess FeLV prevalence in free-roaming stray cats in Switzerland. As part of a joint project between the Network Animal Protection (NetAP) in Switzerland and the Clinical Laboratory (Clin Lab) of the Vetsuisse Faculty at the University of Zurich, stray cats were tested for FeLV (Clin Lab) when they were captured during neutering campaigns (NetAP). The results were not individually assessed for each cat, as making decisions for positive cases poses challenges from an animal welfare standpoint. Instead, the results were analyzed on a per-site or per-area basis. These aggregated data were integrated into a map illustrating FeLV prevalence among free-roaming cats in different geographic areas of Switzerland. The chosen approach enabled the identification of problematic regions where it is advisable to consider the FeLV vaccination of cats as essential. 

## 2. Materials and Methods

### 2.1. Design of the Study

This prospective study employed opportunistic sampling. We included saliva samples collected from cats subjected to a trap–neuter–return program (NetAP, Esslingen, Switzerland). The study was conducted in full compliance with Swiss laws and after it was confirmed that no animal study permit was required. The saliva swab sampling was non-invasive, with the samples collected while the cats were under general anesthesia, which was administered as part of the neutering process. The study results did not affect the outcome for the cats. Following a suitable monitoring period under veterinary supervision and ensuring the cats had fully recuperated from the operation, all cats were returned. The sample collection continued for more than four years, spanning from February 2019 to June 2023.

### 2.2. Sample Collection

Saliva swabs were collected by attending veterinarians, resulting in 1719 samplings. Eight samples were excluded due to duplication during the same sampling event (*n* = 3), samples missing in transit via mail (only the envelope and data sheet arrived; *n* = 4), or sampling from a cat residing abroad (*n* = 1). Seven samples lacked information about their origin within Switzerland, and for 12 samples, only the Swiss canton was known, not the precise sampling location. Thus, 1711 samples were included in the study.

Veterinarians involved in the trap–neuter–return program were equipped with the necessary materials, which included screw-cap tubes (1.5 mL, Sarstedt, Nümbrecht, Germany) and cotton swabs with plastic shafts (M-Budget, Migros Genossenschafts-Bund, Zurich, Switzerland), along with detailed instructions outlining the correct swabbing procedure and prepaid return address labels. The veterinarians were instructed to collect samples from all stray cats undergoing neutering within the program without consideration of the animal’s age, sex, vaccination status, or health condition. The swab was to be gently rubbed along the cat’s cheek pouches and under its tongue and placed in a tube, and the external tip of the swab was removed before sealing the tube. Subsequently, the samples were dispatched via postal mail at ambient temperature.

### 2.3. Data Collection for Prevalence Study

For each cat sampled, a concise questionnaire was filled out by the attending veterinarian. This questionnaire included details such as the sample ID, sampling date, attending veterinarian’s name, postal address with the area code, where the cat was caught, approximate age and sex of the cat, and the outcomes of the physical examination (categorized as healthy or sick, with occasional specification of the major clinical issue when the cat was unwell).

### 2.4. Sample Preparation and Molecular Analysis

The sample processing followed the previously described procedure [3]. In summary, the samples were first resuspended in 200 µL Hank’s Balanced Salt Solution (HBSS) through vortexing, followed by incubation at 42 °C for 10 min. The tubes were subsequently centrifuged at 8000× *g* for 1 min to eliminate any residual liquid from the inner surface of the lid. The swabs were then inverted, the tubes were centrifuged once more, and the swabs were removed from the tubes containing the liquid sample material, which was then stored at −80 °C for subsequent use. The liquid samples were then pooled using a pipetting robot, with up to 96 samples being combined in 20 pools. For additional information, refer to Studer et al., 2019 [3].

The total nucleic acid (TNA) was extracted from 200 µL of the sample pools using the MagNA Pure LC Total Nucleic Acid Kit—High Performance and the MagNA Pure LC instrument (Roche Diagnostics, Mannheim, Germany). Two negative controls containing phosphate-buffered saline (PBS) were simultaneously prepared with every batch of samples to oversee and detect any potential cross-contamination.

FeLV viral RNA was detected using 5 μL of TNA, and a previously described real-time TaqMan FeLV RT-qPCR [11] on an ABI PRISM 7500 Fast Sequence Detection System (Applied Biosystems, Foster City, CA, USA) with some modifications, as described previously [3]. Each PCR run included positive controls (RNA standard template) [11] and negative controls (PBS) to ensure accuracy and reliability.

The pooling method facilitated the pinpointing of individual samples that might have played a role in generating the positive pool outcomes. From each of these individual samples, TNA was extracted using 150 μL of the original liquid sample material, and subsequently, FeLV real-time RT-qPCR was conducted according to the previously described procedure. The FeLV input copy numbers in the individual samples were calculated after amplifying 10-fold serial dilutions of an RNA standard template, following the described method [11]. Subsequent analyses were conducted using the FeLV RT-qPCR results obtained from individual samples. FeLV viral RNA loads were classified as “high” (>10^6^ copies/PCR) or “low” (≤10^6^ copies/PCR).

### 2.5. Statistical Analysis

All data were compiled and analyzed using Excel (Microsoft, Wallisellen, Switzerland) and GraphPad Prism software version 10.1.1 (GraphPad, San Diego, CA, USA). FeLV prevalence (as determined with RT-qPCR from saliva as a measure for antigenemia) in sick and healthy cats, female and male cats, and adult cats and kittens, and frequencies of viral RNA loads, classified as “high” and “low” loads, were analyzed using the chi-square test (p_Chi_) or Fisher’s exact test (p_F_). Odds ratios (ORs) and 95% confidence intervals (CIs) are given for frequencies. Visualization of the data was performed using the Quantum Geographic Information System (QGIS 3.6.11—Hannover version, Open Source Geospatial Foundation Project. http://qgis.org), and *p*-values of <0.05 were considered statistically significant.

## 3. Results

### 3.1. Sample Size and Characteristics

We obtained 1711 saliva samples from cats residing in 19 out of the 26 Swiss cantons. Out of the 1711 cats, 973 (57%) were female, 726 (42%) were male, and reliable sex information was lacking for 12 cats (Table 1). Notably, 1335 (78%) were considered in good general health or clinically healthy, while 299 (18%) were sick at the time of examination. Information regarding the health status of 77 cats was unavailable. 

The majority of the cats were considered adults (1269; 74%): 715 were young adults (age 1 to ≤6 years), 545 were considered “adults” (age not specified), and 9 were mature adults (age > 6 to <10 years; Table 1). About one-fifth of the cats included were kittens (363; 21%): 134 were young kittens (age up to 6 months), 190 were older kittens (age > 6 months <1 year), and 39 were just considered “young” by the attending veterinarians. Twenty-three cats were seniors (>10 years of age; 1.3%). Information concerning the ages of 56 cats was not reported. For statistical analyses, all adults were combined into one group, and all kittens and “young” cats were combined into another group; the reasoning behind this is that, for many stray cats, only an estimation of age could be given, not an exact age.

### 3.2. FeLV Sample Prevalence and Geographic Distribution

Overall, the FeLV prevalence in the 1711 stray cats was 4.0% (95% CI 3.1–5.0%). This is significantly higher than what has been reported using identical methods in saliva samples from 300 privately owned cats presented to veterinarians in Switzerland in 2012/13 (5/300; 1.7%, 95% CI 0.5–3.8%; p_F_ = 0.0454; OR 2.4, 95% CI 1.0–6.1%, Study 1 [2]) and using blood samples from 881 cats tested for free FeLV p27 antigen in 2013 to 2016 (18/881; 2.0%, 95% CI 1.2–3.2%; p_F_ = 0.0104; OR 2.0, 95% CI 1.2–3.4%, Study 2 [2]). However, it is not statistically different from the most recent available data in privately owned cats presented to veterinarians from 2016/2017, where saliva samples from 7/290 Swiss cats were FeLV RT-qPCR positive (2.4%, 95% CI 1.0–4.9%; pan-European FeLV study [3]). 

Only for cantons with more than 20 samples, the FeLV sample prevalence was calculated (Table 2). Particularly high sample prevalences were found in the cantons of St. Gall (21.9%; 95% CI 12.5–34.0%), Thurgau (10.5%; 95% CI 5.9–17.0), and Lucerne (7.6%; 5.1–10.7). These prevalences were significantly higher than the prevalence in the remaining Swiss stray cats (p_F_ < 0.0006). In the canton Neuchâtel (4.3%), the prevalence corresponded approximately to the overall prevalence in all stray cats (4.0%), while in all other remaining sampled cantons, the prevalence was lower (Table 2). 

In most towns (16/19 area codes with positive samples), only one or two cats were FeLV positive (Table 3 and Table A1). In the remaining three towns in three distinct areas of Switzerland (Lucerne, St. Gall, and Thurgau), a high number of the sampled cats were positive. 

**Figure 1:**
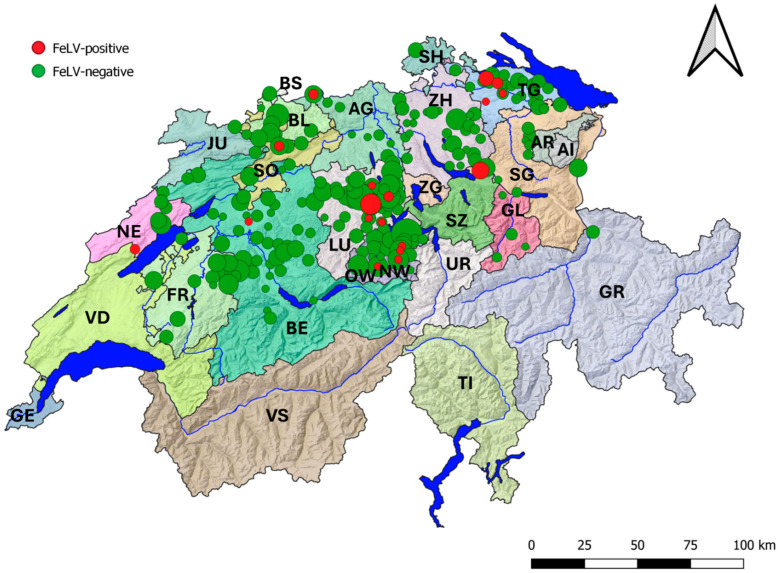
Origins and FeLV RT-qPCR results of all samples collected from free-roaming stray cats in Switzerland during the trap–neuter–return program. Sample collection depended on the areas, where the program was performed. Viral RNA was detected by RT-qPCR in saliva samples as a measure for antigenemia [10]. FeLV-negative samples are shown as green dots; FeLV-positive samples are depicted as red dots. The size of the dots is proportional to the number of cats sampled at the concerned location. Several hotspots with high infection rates were observed, particularly in Central Switzerland (cantons Lucerne (LU), in Nidwalden (NW), in Obwalden (OW)), at the lake of Zurich (canton St. Gall, SG), and in the Thurgau (TG). For the other abbreviations of the canton names, see Table 2.

In a town in the canton Lucerne, close to the lake of Sempach (area code 6206, Figure 1), almost every second cat tested FeLV positive (23/51; 45%). The cats lived at four different locations within the town (seven locations sampled); there were 4 FeLV-positive kittens and 19 FeLV-positive adult cats. Fourteen animals (three kittens and eleven adults) were reported to be sick (mainly cat flu, flea infestation, worms, and bad teeth). 

In the canton St. Gall, the situation was somewhat different. The 14 FeLV-positive cats lived at one site in a town (area code 8646). At this site, 14 out of 20 cats tested positive (70%). There were 2 kittens and 12 adult cats. Most of the cats were healthy (both kittens and 10 adults). 

A similar situation was found in a town in the canton of Thurgau (area code 8506), where 13 cats from several sites were sampled. However, 9 FeLV-positive cats out of the 13 sampled cats (70%) all lived at one site (9/9 cats were FeLV positive at that site). They were all adults and healthy. The four FeLV-negative cats in this town lived at two other sites (maximum distance of 3.5 km): 4/4 tested were negative. Still, it was a very limited hotspot. 

The geographic distribution of all samples collected within this study and of FeLV-positive samples is given in Figure 1.

### 3.3. FeLV Sample Prevalence and Sex, Age, and Health Status of the Cats

Almost exclusively sexually intact cats were included in the study since the cats were subjected to a trap–neuter–return program. Intact male cats were FeLV positive twice as often (41/726; 5.6%) compared to female intact cats (27/973; 2.8%; p_F_ = 0.0037; Table 4). 

Adult cats (≥1 and <10 years of age; 59/1268; 4.6%) were FeLV positive 2.5 times more often than younger cats/kittens (7/363; 1.9%; p_F_ = 0.0223). The oldest FeLV-positive cat was a healthy intact female of about 10 years of age living in the canton Solothurn. It was one of two cats that tested positive within this area (same area code). The youngest FeLV-positive cats were three animals of about 6 months of age; all three were from the same location, and two were sick with cat flu.

Sick cats were FeLV positive 2.4 times more often (23/299; 7.7%) compared to healthy cats (44/1335; 3.3%; p_F_ = 0.0018). FeLV-positive cats predominantly showed diseases not commonly primarily related to an FeLV infection: cat flu (*n* = 9); parasites, including fleas, worms, and mites (*n* = 6); eye conditions (*n* = 1); dental problems (*n* = 9); and low body weight (*n* = 1). For four cats, no details were given on clinical signs.

### 3.4. Viral Loads

The viral load was determined semi-quantitatively in saliva samples using RT-qPCR. “High” viral loads (>10^6^ copies/PCR) were found in 22 samples and “low” loads (≤10^6^ copies/PCR) in 46 samples. 

There was a tendency for sick cats to more frequently have “high” loads (11/23; 48%, 95% CI 27–70%) compared to healthy cats (10/44; 23%, 95% CI 12–38%; pF = 0.522). There was no difference in the viral loads between different sexes (male versus female), ages (kittens vs. adult cats), or origins of the cats (PLZ). The highest loads (1.0 × 10^8^ and 2.3 × 10^8^ copies/PCR) were found in two female young adult cats, one sick and one without information concerning its health status. Both were living in the same location. No further cats were tested from this location.

### 3.5. FeLV-Positive Cats in Switzerland from 2012 to 2023

**Figure 2:**
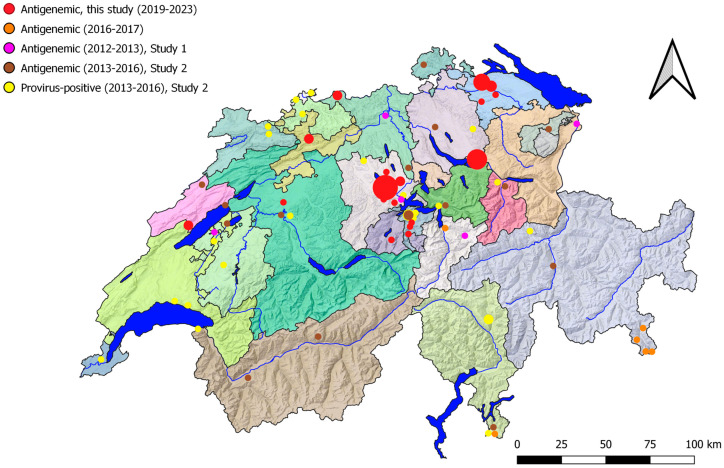
Comparison of the origins of the FeLV-positive samples detected within the current and three previous studies in Switzerland: stray cats, this study, antigenemic* cats (red); pan-European FeLV study [3], antigenemic* cats, 7 of 290 positive (orange); Study 1 [2], antigenemic* cats, 5/300 positive (violet); Study 2 [2], antigenemic cats, 18/881 positive (brown) and only provirus-positive cats, 29/881 (yellow). The size of the dots is proportional to the number of cats sampled at the concerned location. Samples that tested negative are not indicated to prevent overloading the map. For the other abbreviations of the canton names see Table 2. * Determination of salivary RNA as a measure for antigenemia.

We compared the geographic distribution of FeLV in stray cats with that of previous studies from Switzerland in mostly privately owned cats presented to veterinarians [2,3] (Figure 2). In Central Switzerland, where a big hotspot of FeLV was found in stray cats, privately owned cats had also been reported to be FeLV infected. Additionally, FeLV-positive stray or owned cats have also been documented in other areas and 24/26 cantons (Table 5).

## 4. Discussion

The identification of FeLV-infected cats is essential for preventing new FeLV infections in naive cats even though efficacious FeLV vaccines are available worldwide. The European Advisory Board on Cat Diseases (ABCD) and the American Association of Feline Practitioners (AAFP) recommend that the retrovirus status of every cat at risk of infection should be known [1,4]. This includes all cats with outdoor access or living outdoors, particularly stray cats. 

The current study is one of the largest studies on FeLV infections in stray cats subjected to a trap–neuter–return program, including a total of 1711 cats. To the best of our knowledge, there have only been two other large trap–neuter studies with more than 1000 cats (Table 6). One was performed in the U.S. and included 733 and 1143 unowned free-roaming cats subjected to a trap(–neuter–return) program in two cities in North Carolina and Florida between 1995 and 2000 [12]. The second one included 1008 cats admitted to a neutering unit in Western Turkey; the study was published in 2018, but we could not find a statement about when the cats had been sampled [13]. For both of these studies, no detailed geographic information was presented.

In the present study, the overall prevalence of FeLV-positive cats (viral RNA in saliva as a measure for antigenemia) was 4.0% (95% CI 3.1–5.0%), with an even higher infection rate in sick cats (7.7%; 95% CI 4.9–11.3%). However, 3.3% (95% CI 2.4–4.4%) of the healthy cats were also FeLV positive. Thus, it is crucial to recognize that even seemingly healthy stray cats must be considered as potential virus shedders. The insights gleaned from this study play a pivotal role in ensuring the well-being of both stray cats and privately owned pets with outdoor access. Furthermore, our data serve as valuable information for educating veterinarians and cat owners about the significance of testing cats for an FeLV infection and the importance of FeLV vaccination.

An FeLV infection was detected by testing saliva samples for viral RNA using RT-qPCR. There is an almost perfect agreement between results from FeLVp27 antigen testing from blood samples and salivary viral RNA [10]. Both methods primarily identify cats with a progressive infection, but they also identify a part of the regressively infected cats in the very early phase of the infection when these cats can also be antigenemic/salivary viral RNA positive. Saliva samples were selected over blood since their collection is less invasive and time-consuming. Furthermore, the use of saliva allows for pooling in screening procedures using sensitive RT-qPCR without compromising sensitivity [10], and it reduces the costs of testing. This becomes particularly significant when dealing with a substantial number of samples. The cats that test positive with RT-qPCR must be assumed to be FeLV shedders, posing an infection risk to other unvaccinated cats.

The FeLV prevalence has decreased in Switzerland for many years due to the improved knowledge of its pathogenesis, the awareness of veterinarians, the availability of excellent diagnostics, and efficacious FeLV vaccines [2]. However, subsequently, FeLV prevalence stagnated at a certain percent, and in the last study performed on cats presented to veterinarians in 2016/17 [3], the prevalence was not any lower than what we have now found in the stray cat population. 

It can now be speculated that this lack of further reduction of FeLV prevalence in privately owned cats is due at least in part to the presence of FeLV in stray and feral cats in many regions of Switzerland as well as the presence of unvaccinated cat populations. The latter includes most of the stray cats as well as unvaccinated owned cats with outdoor access. In the Swiss study in 2016/2017 [3], only 24% of the 290 Swiss pet cats presented to veterinarians were indoor-only cats (personal communication R. H.-L.). On the positive side, the majority of the cats with outdoor access (157/215; 73%) had been vaccinated against FeLV. Still, this leaves more than one in four client-owned cats (27%) presented to veterinarians in Switzerland with outdoor access but no protection against FeLV by vaccination; most of these cats were more than 13 weeks old, so they should have received some vaccination [3] (and personal communication R. H.-L.).

A positive aspect of the present study is that the locations where the cats were trapped/lived were recorded for almost all animals, not only the overall prevalence for all of Switzerland. As a result, the data from this study are useful for planning specific measures in locations with identified FeLV issues. There were several recognized geographical hotspots for FeLV infections in Switzerland, and they presented differently. The biggest hotspot was located in the canton of Lucerne (area code 6206). The infection was quite widespread within the town in that cats from several sites tested FeLV positive. Thus, in this area, veterinarians and cat owners should be particularly vigilant and vaccinate every cat that has outdoor access without exception. In the canton of St. Gallen (area code 8646), it might be most effective to address the problem at the one identified site within the town, where 14/20 tested cats were FeLV positive. Moreover, all cats, including stray and privately owned cats that are free-roaming in proximity to this site, should be tested and vaccinated. A similar situation was found in a town in the canton of Thurgau, where a very limited hotspot was detected within a town (area code 8506).

We cannot exclude further FeLV hotspots of stray cats in Switzerland. Indeed, there may be additional hotspots in areas not sampled in the current study. For example, in a study investigating the presence of feline gammaherpesvirus by means of PCR in 91 stray cats from the Swiss canton of Jura, 7 cats (7.7%) tested positive for FeLV provirus [14], while in the current study, the 8 tested cats from Jura were FeLV negative. No serum was available from the cats in the earlier study to test for FeLV p27 antigen [14]. To recognize hotspots in all areas in Switzerland, a more systematic sampling and an even larger number of cats would be needed. This was not possible due to organizational, financial, and time restraints. However, there were also widely scattered single FeLV-positive cats in the current study. Moreover, when revisiting studies performed during the last ten years in Switzerland on privately owned cats [2,3], it is recognized that FeLV is present in every region of Switzerland (Table 5 and Figure 2). 

Other studies on the occurrence of FeLV infections in stray and/or feral cats in other countries have been performed, and FeLV prevalence has varied widely among the different studies, from 0 to 24% (Table 6). Several of the studies, particularly those including a high number of cats, have been retrospective studies relying on shelters and clinics to provide test results on stray cats or cats surrendered to the shelters. Therefore, these data are not directly comparable to the research including cats in trap–neuter–return programs. The latter most often include cats that would otherwise never have received veterinary treatment, and many of these cats would be unlikely to be adopted by most people. In addition to the targeted population of cats, several other factors influence the reported FeLV prevalence. For example, these include the method of detection of FeLV. Table 6 only gives FeLV prevalences for studies that tested for antigenemia. Moreover, FeLV prevalence varies among different countries. For example, FeLV prevalence was found to be higher in Southern European countries compared to Northern European countries [3]. Moreover, the investigated timeframe varied among the different studies, and variation in FeLV prevalence over time has been reported [15]. In addition, factors that have been identified in one study in Korea, which positively associated FeLV prevalence with urban stray cats, are the degree of supplemental feeding and cat caretaker activity; the higher the human food provision and care for stray cats, the higher the FeLV prevalence in stray cats [16]. The authors speculated that an increased FeLV prevalence was found due to an increased gathering of cats around food provisioned by the cat caretakers. In our study, we had no information on the degree of caretaker activity and feeding of the stray cats. Finally, from our data, we speculate that the investigated geographic area within a country or a smaller geographic area may have an influence on FeLV prevalence since hotspots, as detected in our study, may exist also in other countries. Indeed, in the large pan-European study, we found evidence of geographic hotspots, e.g., in Ireland, where five out of seven FeLV-positive cats originated from two veterinary facilities in Galway and Kerry [3]. The large differences in FeLV prevalence in different populations show that studies are needed for individual countries and regions.

**Table 6:** FeLV prevalence studies in stray cats from different countries.

Country	Year	Number of Tested Cats	FeLV Prevalence (%) ^1^	Specifics on Tested Cats	Reference
**Americas**					
Canada (Ottawa)	2001–2003	74	6.7	Stray cats were selected based on risk factors for FIV infection	[17]
Canada	2007	1556	2.7	Animal shelters providing test results on stray cats	[18]
USA, Canada	2004	4550	1.6	Animal shelters providing test results on stray cats	[6]
USA, Canada	2010	23,560	3.2	Animal shelters and clinics providing test results on unowned cats (stray, feral, or owner-relinquished)	[19]
USA (CA)	2001–2003 2005–2007 2012–2013	1848 1918 709	1.7 1.1 0.3	Retrospective analysis of records on feral cats	[20]
USA (NC, FL)	1995/1996/1998–2000	733/1143	5.3/3.7	TNR program; at one site, FIV- or FeLV-positive cats were euthanized	[12]
USA (Hawaii)	2002–2004	68	16.2	Capture of feral cats	[21]
Grenada, West Indies	2004–2007	101	0	Capture and neutering of feral cats	[22]
Brazil	2012–2013	30	0	Zoonosis control center	[23]
**Europe**					
UK	1997	517	3.5	Stray cats picked up by RSPCA	[24]
UK	2011–2012	726	2.3	Two rehoming centers including strays	[25]
Ireland	2007–2008	86	0.0	Health check and homing	[26]
Finland (Helsinki)	1990	196	1.0	Free-roaming cats	[27]
Belgium	1998–2002	346	3.8	T(NR) program ^2^	[28]
Belgium	Not stated	130	4.6	TNR program	[29]
Belgium	2010–2012	302	0.7	TNR program, selective removal of FIV-positive cats	[30]
Denmark	2006–2009	7255	0–0.9	Stray and owned cats found in distress	[31]
Netherlands	2020–2022	580	0	Stray cats	[32]
France	2007	492	1.0	15 rural populations of owned and unowned cats	[33]
Northern Italy	2008–2010	316	3.8	TNR program	[34]
Northern Italy	2014	90	6.1	TNR program	[35]
Southern Italy	2014–2023	1322	7.6	Stray and owned cats	[36]
Italy	2017–2023	314	6.0	Stray and owned cats	[37]
Spain (Catalonia)	2012	116	6.0	Cats in shelters	[38]
Spain	2014–2017	632	6.3	TNR program	[39]
Spain (Madrid)	2012–2013	346	4.0	Health control program, TNR	[40]
Spain (Zaragoza)	2020	114	4.4	Testing for SARS-CoV-2	[41]
Spain (Zaragoza)	2020–2022	254	3.2	TNR program	[42]
Spain (Zaragoza)	Not stated	178	4.5	TNR program	[43]
Portugal (Lisbon)	2003–2005	198	7.1	TNR program	[44]
Greece	2013–2016	123	4.2	Stray cats brought in for neutering and treatment	[45]
Western Turkey	Not stated	1008	3.3	Female stray cats admitted to neutering unit	[13]
**Asia**					
Taiwan	1993–1994	75	1.3	Cats in shelters	[46]
Korea (Seoul)	2014–2015	276	23.2	TNR program	[16]
China	2014–2015	260	12.0	Stray cats	[47]
**Oceania**					
Western Australia	2011–2013	2151	1.0	Cats surrendered to two shelters	[48]
Australia (Sidney)	2013/2015	38/51	18/24	Cats in two shelters with group housing	[49]
New Zealand	2014	237	0.8	Cats in shelter	[50]

^1^ Detection of FeLV p27 antigen. In some studies, PCR was used, and none of the cats were positive; thus, it is assumed that all cats also tested negative for FeLV p27 antigen, and the study was also included. ^2^ Only healthy cats were neutered. Cats in bad general condition were euthanized. TNR = trap–neuter–return; RSPCA = Royal Society for the Prevention of Cruelty to Animals; FIV = feline immunodeficiency virus.

Our study exclusively identified cats with antigenemia, determined through the presence of viral RNA in saliva. The detection of antigenemia is what most frequently is carried out in FeLV prevalence studies since it can be easily assessed using point-of-care tests. It is important to note that positive p27 antigen point-of-care tests should be confirmed ideally using provirus PCR, particularly in situations where the cat cannot be assigned to a FeLV high-risk group, including cats from FeLV-positive environments or those exhibiting clinical signs commonly associated with FeLV [1,4]. Due to the unavailability of blood samples, we could not test for FeLV provirus with qPCR. Consequently, stray cats with a regressive FeLV infection, provirus PCR positive but antigen negative [7,8], were not detected in the current study. In a previous study in Switzerland, 2.2% of the 881 tested cats were antigenemic and had presumably progressive FeLV infections, while a total of 5.3% were provirus positive, encompassing progressively and regressively infected cats [2]. An even higher number of cats must be assumed to be exposed to FeLV. This includes not just progressively and regressively infected cats and some rare focal infections but also cats with an abortive infection outcome [1,4]. The latter cats exhibit strong immunity, preventing FeLV provirus integration into their cells. This type of infection is not detectable with FeLV antigen or PCR detection test methods, which remain negative [7,8]. However, the detection of antibodies to FeLV is supposed to also recognize cats with an abortive FeLV infection [51]. The overall prevalence of FeLV, including progressive, regressive, focal, and abortive infections, was about 21% for Italy and Portugal and also 9% for France and Germany [52]. Notably, the latter had previously been assumed to have a very low FeLV prevalence when considering only antigenemic cats [3,5].

Remarkably, a high number of FeLV-positive cats was considered healthy or in good general health by the attending veterinarians. Only a minority (18%) were reported to be sick or in poor general health by the attending veterinarians. However, it was not the goal of the present study to investigate clinical signs in the sampled cats, and thus, the study setup was not designed for this. Clinical signs were only occasionally reported and not according to an exact protocol; thus, the reported data largely depended on the attending veterinarian. Nonetheless, the large number of cats reported to be in good health is promising and positive for cats’ welfare.

This study included almost exclusively sexually intact cats, and our data confirm that intact male cats are more often FeLV positive than intact female cats. Increased FeLV infection rates in intact and male cats have been reported previously [3,6]. Potential reasons for the higher prevalence in male cats include a larger territory of tomcats and more aggressive interactions than in female cats [53]. This further confirms that, for FeLV and for not only feline immunodeficiency virus (FIV), aggressive interactions are an important means of virus transmission. This should not be forgotten, even though FeLV can also be transmitted by social interaction, including sharing food and water dishes and mutual grooming [4]. The latter was assumed to be a factor in the study in Korea, where female cats were more often FeLV infected, with an increased infection prevalence in areas with increased care for and feeding of stray cats [54]. The authors suspected the mechanism is not primarily the population density but rather the changed behavior of cats in the presence of safe food sources; this can affect cats’ exposure to a pathogen such as FeLV, which is transmitted directly by social contact.

Another factor that was associated with increased risk for an FeLV infection was adult age. Cats older than one year were more often FeLV infected than kittens. Our data further confirm those of other studies, where adult cats have been more often FeLV positive than kittens and young cats [3,18,25]. The explanation for stray cats may be somewhat different than what is suspected for client-owned cats. For the latter, it is assumed that even when FeLV positive, these cats may live longer since the infection is recognized early due to increased awareness and the early onset of excellent medical supportive care [4]. In stray cats, it can be hypothesized that, if young cats that are highly susceptible to a progressive FeLV infection encounter the virus in the absence of any medical care but with the presence of potential co-infections and under suboptimal living conditions, they may succumb early due to FeLV-associated fatal disease; thus, they may die before they could enter trap–neuter–return programs.

The viral loads were determined semi-quantitatively in saliva samples, as the precise measure was hindered by the uncertainty of the collected volume of saliva and the biological dilution of the sample. However, very high loads were found in some cats (>10^8^ copies/PCR) not reported previously in 141 FeLV RT-qPCR positive cats detected during the pan-European FeLV study in 32 countries. Further studies should include the molecular characterization of this particular FeLV isolate.

A major opportunity, but also limitation, of this study is the sample collection, which was performed purely opportunistically and depended on the trap–neuter–return program of NetAP. While this was an excellent opportunity to receive samples from stray cats, which otherwise cannot be sampled, the sampling procedure could not be adapted to the goals of this study. The primary goal of the program was to neuter cats, which is the most important for reducing the number of future stray cats. In five cantons, we received fewer than ten samples. And from seven cantons, we did not receive any samples for this study. Therefore, the situation concerning FeLV infections in stray cats in the southern cantons of Switzerland could not be assessed. 

After having conducted this study, one question that remains is what should be done with FeLV-positive cats. The 68 FeLV-positive cats in the current study were released back into the natural environment after they had been neutered and had received basic veterinary care; none of the cats were euthanized. In contrast, in another study conducted in the U.S. two decades ago, FeLV-positive stray cats were euthanized independently of their health status [12]. In a more recent Belgian study on FIV, the selective removal of FIV-infected cats during a trap–neuter–return campaign was demonstrated to have a drastic effect on FIV prevalence: within three years, the prevalence in the remaining population decreased from 31% to 13% FIV-positive cats [30]. In a recent survey in cat shelters in Florida, FeLV testing during a trap–neuter–return campaign was the least common; only 18% of the shelters tested cats for FeLV during these programs [55]. Testing was more common prior to the adoption or transfer of cats. Most shelters aimed to rehome FeLV-positive cats by adoption; nonetheless, 43% of the shelters also performed the euthanasia of FeLV-positive cats. Historically, routine testing for FeLV in Swiss trap–neuter programs had not been implemented; it was only introduced for the purposes of the current study. The primary objective of the present study did not involve the individual identification of FeLV-positive cats, as their removal was ethically precluded. However, trap–neuter–return programs, implemented for decades, have demonstrated the potential to reduce feline populations and enhance overall population welfare; this is evidenced by an increased average age of the population and a decrease in retrovirus prevalence [15]. Additional measures to reduce FeLV prevalence in stray cat populations should include FeLV vaccination. In a colony of 30 cats naturally exposed to FeLV in Pisa, Italy, vaccination was effective in protecting (FIV-negative) cats [56]. The authors of the Pisa study were able to conduct this study thanks to their direct involvement in providing care and sustenance for the colony of semi-domesticated cats. It remains to be seen how the results of that study can be transferred to stray cat populations in general, where cats have little or no contact with humans. 

## 5. Conclusions

We hypothesize that this epidemic situation with FeLV hotspots in certain regions of the country is not limited to Switzerland, and therefore, we strongly recommend similar investigations in other countries where FeLV prevalence has failed to further decrease.

On the other hand, we found no area of Switzerland that can be assumed to be without an FeLV infection risk for free-roaming cats. Moreover, cats are relocated frequently in Switzerland, and most cats do go outside in this country. Thus, we highly recommend vaccinating all cats against FeLV, with few exceptions (such as lifelong indoor-only cats without any contact with other cats of uncertain FeLV status). 

In future trap–neuter–return programs, it is suggested that free-roaming stray cats, particularly in problematic areas with high FeLV infection rates, are vaccinated to gradually reduce the FeLV prevalence in these regions over time by reducing the number of susceptible cats. However, all currently available FeLV vaccines ask for two vaccinations for protective immunity against FeLV. This might seriously hamper the vaccination of cats unaccustomed to human interaction. 

## Figures and Tables

**Table 1:** Sample characteristics (all cats and FeLV-positive cats).

Parameter	Categories	All Cats (*n* = 1711)	FeLV-Positive Cats ** (*n* = 68)
Sex	Female intact	973 (57%)	27 (40%)
	Male intact	726 (42%) *	41 (60%)
	Unknown	12 (0.7%)	0 (0%)
Age	Kittens (<1 year)	363 (21%)	7 (10%)
	Young adults (1 to ≤6 years)	715 (42%)	32 (47%)
	Mature adults (6 to <10 years)	9 (0.5%)	1 (1.5%)
	“Adults” (no age specified)	545 (32%)	26 (38%)
	Senior (≥10 years)	23 (1.3%)	1 (1.5%)
	Unknown	56 (3.3%)	1 (1.5%)
Health	Healthy	1335 (78%)	44 (65%)
	Sick	299 (17%)	23 (34%)
	Unknown	77 (4.5%)	1 (1.5%)

* One cat was a castrated male. ** FeLV positivity was determined using RT-qPCR from saliva as a measure for antigenemia.

**Table 2:** Number of samples included in this study and canton distribution, number of FeLV-positive samples, and sample prevalence in the different cantons, with over 20 samples submitted.

Swiss Canton		Number of Samples Included	No. of FeLV-pos.	FeLV Prevalence % (95% CI) ^1^
AG	Aargau	89	2	2.2 (3.0–7.9)
AR	Appenzell Inner-Rhodes	1	0	
BE	Bern	276	1	0.4 (0.0–2.0)
BL	Basel-Country	36	0	0.0 (0.0–9.7)
BS	Basel-City	15	0	
FR	Fribourg	94	0	0.0 (0.0–3.8)
GL	Glarus	8	0	
GR	Grisons	6	0	
JU	Jura	8	0	
**LU**	**Lucerne**	**382**	**29**	**7.6 (5.1–10.7)**
NE	Neuchâtel	47	2	4.3 (0.5–14.5)
NW	Nidwalden	157	2	1.3 (0.2–4.5)
OW	Obwalden	116	2	1.7 (0.2–6.1)
**SG**	**St. Gall**	**72**	**14**	**21.9 (12.5–34.0)**
SH	Schaffhausen	3	0	
SO	Solothurn	124	2	1.6 (0.2–5.7)
**TG**	**Thurgau**	133 *	**14**	**10.5 (5.9–17.0)**
VD	Vaud	21	0	0.0 (0.0–16.1)
ZH	Zurich	116	0	0.0 (0.0–3.1)
	Canton unknown	7	0	
Total		1711	68	4.0 (3.1–5.0)

* Area code unknown for 12 cats sampled in the canton Thurgau. ^1^ Percentages and 95% confidence intervals (CIs) were calculated only for cantons with more than 20 samples available. Cantons with prevalences higher than the average prevalence are highlighted in bold.

**Table 3:** Area codes of all locations where FeLV-positive cats were found, with number of samples collected and number of FeLV-positive cats at that location.

Area Code	Canton	Number of Samples Included	No. of FeLV-pos.
2027	NE	3	2
3053	BE	1	1
4313	AG	17	2
4719	SO	18	2
6010	LU	4	1
6025	LU	10	1
6072	OW	23	1
6102	LU	9	1
6203	LU	10	1
**6206**	**LU**	**51**	**23**
6274	LU	34	2
6383	NW	20	1
6386	NW	26	1
6388	OW	25	1
**8506**	**TG**	**13**	**9**
8514	TG	5	1
8555	TG	5	3
**8646**	**SG**	**20**	**14**
9548	TG	1	1

Data from areas with prevalences higher than the average prevalence and more than 10 samples tested are highlighted in bold.

**Table 4:** Sample characteristics: sex, age, and health status.

Parameter	Categories	FeLV-Positive/ Tested	FeLV Prevalence % (95% CI)	Odds Ratio ^1^ (95% CI)	p_F_-Value ^1^
Sex	Intact female	27/973	2.8 (1.8–4.0)	Ref.	
	Intact male	41/726	5.6 (4.1–7.6)	2.1 (1.3–3.4)	0.0037
	Unknown	0/18			
Age	Kittens (<1 year)	7/363	1.9 (0.8–3.9)	Ref.	
	Adults (1 to <10 years)	59/1269	4.6 (3.6–6.0)	2.5 (1.1–5.5)	0.0223
	Senior (≥10 years)	1/23			
	Unknown	1/56			
Health	Healthy	44/1335	3.3 (2.4–4.4)	Ref.	
	Sick	23/299	7.7 (4.9–11.3)	2.4 (1.5–4.2)	0.0018
	Unknown	1/77			

^1^ Odds ratio (OR) and *p*-value Fisher’s exact were calculated from sick vs. healthy, with sick cats being infected more often; kittens vs. adults, with adults being infected more often; and females vs. males, with males being infected more often. The number of seniors was not included because of this category’s low number of cats. Ref. = reference. CI = confidence interval.

**Table 5:** Summary of this and previous studies [2,3]: FeLV-infected cats were observed in the 26 Swiss cantons. Of note, not all cantons were sampled with the same frequency.

Canton	Number of FeLV-Positive Stray Cats ^1^	Number of FeLV-Positive Cats Presented to Veterinarians ^2^
AG	2	2
AR		1
AI		
BE	1	2
BL		2
BS		1
FR		1
GE		1
GL		2
GR		6
JU		2
LU	29	4
NE	2	2
NW	2	8
OW	2	1
SG	14	2
SH		1
SO	2	0
SZ		2
TG	13	1
TI		6
UR		2
VD		5
VS		3
ZG		
ZH	1	2
Total	68	59

^1^ This study; 2019–2023. ^2^ Previous studies [2,3]; at least 20 samples were included from each canton; 2012–2017.

## Data Availability

All data are presented within this article.

## Appendix A

**Table A1:** Details on the number of FeLV-positive, FeLV-negative, and total number of tested cats per region (canton/area code). The total for each canton is given in bold. No percentages of FeLV positives were calculated because of small numbers for most regions (area codes).

Canton, Area Code	FeLV-Negative	FeLV-Positive	Total Number of Cats
**AG**	**87**	**2**	**89**
4143	1		1
4313	15	2	17
4324	2		2
5033	1		1
5070	2		2
5462	7		7
5600	1		1
5607	3		3
5620	1		1
5623	7		7
5630	9		9
5637	25		25
5646	13		13
**AR**	**1**		**1**
9103	1		1
**BE**	**275**	**1**	**276**
2616	7		7
2720	10		10
2723	4		4
3043	3		3
3053	0	1	1
3072	6		6
3076	4		4
3088	7		7
3096	15		15
3114	2		2
3125	3		3
3132	6		6
3150	5		5
3152	27		27
3157	3		3
3158	21		21
3183	13		13
3204	11		11
3256	1		1
3270	1		1
3272	3		3
3298	8		8
3303	4		4
3326	1		1
3421	1		1
3423	3		3
3427	9		9
3439	3		3
3507	4		4
3510	13		13
3531	6		6
3532	16		16
3534	13		13
3537	4		4
3553	2		2
3628	1		1
3635	1		1
3674	4		4
3754	1		1
3755	5		5
3800	1		1
4537	1		1
4704	4		4
4942	2		2
4950	16		16
**BL**	**36**		**36**
2814	7		7
4203	13		13
4253	2		2
4438	7		7
4447	7		7
**BS**	**15**		**15**
4055	5		5
4056	8		8
4105	1		1
8425	1		1
**FR**	**94**		**94**
1618	7		7
1628	10		10
1714	47		47
1717	7		7
1725	6		6
1735	1		1
3185	16		16
**GL**	**8**		**8**
8767	1		1
8773	4		4
8783	2		2
8867	1		1
**GR**	**6**		**6**
7302	6		6
**JU**	**8**		**8**
2828	3		3
2829	5		5
**LU**	**353**	**29**	**382**
6004	2		2
6010	3	1	4
6018	13		13
6020	2		2
6022	20		20
6023	3		3
6025	9	1	10
6026	38		38
6032	4		4
6034	17		17
6035	4		4
6043	3		3
6102	8	1	9
6103	4		4
6110	3		3
6113	4		4
6145	20		20
6147	4		4
6156	1		1
6162	8		8
6203	9	1	10
6205	2		2
6206	28	23	51
6207	44		44
6208	11		11
6213	1		1
6216	1		1
6218	1		1
6232	13		13
6247	4		4
6248	7		7
6274	32	2	34
6275	5		5
6276	22		22
6280	3		3
**NE**	**45**	**2**	**47**
2027	1	2	3
2042	22		22
2043	12		12
2054	5		5
2333	5		5
**NW**	**155**	**2**	**157**
6052	5		5
6363	5		5
6370	2		2
6372	13		13
6373	5		5
6374	63		63
6375	1		1
6376	2		2
6382	8		8
6383	19	1	20
6386	25	1	26
6387	7		7
**OW**	**114**	**2**	**116**
6055	16		16
6056	2		2
6060	10		10
6062	1		1
6063	4		4
6064	10		10
6072	22	1	23
6073	1		1
6074	23		23
6388	24	1	25
6390	1		1
**SG**	**58**	**14**	**72**
8646	6	14	20
8717	1		1
8733	17		17
8872	2		2
9113	6		6
9114	1		1
9115	2		2
9230	5		5
9465	16		16
9633	2		2
**SH**	**3**		**3**
8226	3		3
**SO**	**122**	**2**	**124**
2545	17		17
3253	15		15
4143	29		29
4208	4		4
4232	19		19
4247	14		14
4717	8		8
4719	16	2	18
**TG**	**119**	**14**	**133**
8254	4		4
8505	2		2
8506	4	9	13
8507	9		9
8508	5		5
8514	4	1	5
8524	2		2
8535	2		2
8554	2		2
8555	2	3	5
8556	4		4
8558	3		3
8561	2		2
8566	11		11
8573	2		2
8575	2		2
8581	1		1
8582	3		3
8585	8		8
8586	4		4
8595	7		7
9216	2		2
9220	10		10
9225	2		2
9325	8		8
9517	2		2
9548		1	1
Unknown area code	12		12
**VD**	**21**		**21**
1534	16		16
1588	5		5
**ZH**	**116**		**116**
8105	1		1
8108	1		1
8124	1		1
8155	2		2
8165	7		7
8185	1		1
8226	7		7
8310	2		2
8314	25		25
8322	3		3
8340	7		7
8354	7		7
8418	1		1
8426	6		6
8489	4		4
8492	2		2
8494	2		2
8604	2		2
8610	13		13
8620	1		1
8625	5		5
8634	1		1
8635	7		7
8708	2		2
8902	2		2
8909	3		3
8952	1		1
**Canton unknown**	**7**		**7**
Area code unknown	7		7
**Total**	**1643**	**68**	**1711**
